# Challenges and support needs in psychological and physical health among pilots: a qualitative study

**DOI:** 10.3389/fpubh.2024.1351568

**Published:** 2024-04-16

**Authors:** Wen Xu, Yuyan Bao, Lin Zhang, Yunfei Li, Erliang Zhang, Huilun Li, Qingqing Jin, Yan Chen, Qingqing Duan, Feng Shi, Linlin Wang, Ziyang Lu, Xuhua Chen, Qijing Gao, Hangyu Han, Bin Ren, Ya Su, Mi Xiang

**Affiliations:** ^1^International Peace Maternity and Child Health Hospital, School of Medicine, Shanghai Jiao Tong University, Shanghai, China; ^2^Shanghai Key Laboratory of Embryo Original Diseases, Shanghai, China; ^3^Shanghai Jiao Tong University School of Medicine, Shanghai, China; ^4^CAAC East China Aviation Personnel Medical Appraisal Center, Civil Aviation Shanghai Hospital, Shanghai, China; ^5^Department of Epidemiology and Prevention, Center for Clinical Sciences, National Center for Global Health and Medicine, Shinjuku, Japan; ^6^School of Public Health, Shanghai Jiao Tong University, Shanghai, China; ^7^School of Nursing, Shanghai Jiao Tong University, Shanghai, China

**Keywords:** occupational health, mental health, physical health, qualitative study, pilots and cabin crew, COVID-19

## Abstract

**Introduction:**

Physical and mental health problems among pilots affect their working state and impact flight safety. Although pilots’ physical and mental health problems have become increasingly prominent, their health has not been taken seriously. This study aimed to clarify challenges and support needs related to psychological and physical health among pilots to inform development of a more scientific and comprehensive physical and mental health system for civil aviation pilots.

**Methods:**

This qualitative study recruited pilots from nine civil aviation companies. Focus group interviews via an online conference platform were conducted in August 2022. Colaizzi analysis was used to derive themes from the data and explore pilots’ experiences, challenges, and support needs.

**Results:**

The main sub-themes capturing pilots’ psychological and physical health challenges were: (1) imbalance between family life and work; (2) pressure from assessment and physical examination eligibility requirements; (3) pressure from worries about being infected with COVID-19; (4) nutrition deficiency during working hours; (5) changes in eating habits because of the COVID-19 pandemic; (6) sleep deprivation; (7) occupational diseases; (8) lack of support from the company in coping with stress; (9) pilots’ yearly examination standards; (10) support with sports equipment; (11) respecting planned rest time; and (12) isolation periods.

**Discussion:**

The interviewed pilots experienced major psychological pressure from various sources, and their physical health condition was concerning. We offer several suggestions that could be addressed to improve pilots’ physical and mental health. However, more research is needed to compare standard health measures for pilots around the world in order to improve their physical and mental health and contribute to overall aviation safety.

## 1 Introduction

It is known that pilots’ health affects their working state. However, the serious risks posed by pilots’ working conditions, including enclosed cabins that are exposed to ultraviolet radiation at high altitudes for long periods (1) and isolation rooms during the COVID-19 pandemic, mean that pilots’ physical and mental health problems have become increasingly prominent; these health problems may impact flight safety (1–4). Previous studies have reported that compared with well-rested people, pilots who felt tired from lack of sleep thought and reacted more slowly, had more memory problems, and were more likely to make mistakes (5–7). The PlaneCrashInfo.com database shows that approximately 50% of flight accidents are related to pilots’ working state; this proportion has not changed significantly over time. These data confirm that pilots’ physical and mental health impact flight safety by affecting their working state. Therefore, improving pilots’ physical and mental health is necessary to improve aviation safety (7).

Previous studies have shown that common health problems among pilots include abdominal distension, headache, fatigue, and depression, attributable to factors such as smoking, drinking, and irregular diet and sleep (6, 8–10). Increased use of caffeine, alcohol, and drugs cannot prevent deterioration of physical and mental conditions but increases the risk for insomnia and sleepiness, whereas exercise can effectively improve pilots’ physical and mental health (6, 11). Most previous studies on this topic were conducted using questionnaires, which could not accurately capture pilots’ subjective feelings (1–6). Qualitative research in this area is relatively scarce, meaning personalized information about pilots’ feelings remains largely unknown. In addition, most previous studies have focused on physical or psychological evaluations. However, both physical and mental health are important components of overall health, and can influence each other; therefore, a discussion combining physical and mental health may provide a more comprehensive perspective on pilots’ health issues. Unfortunately, many unilateral studies could not effectively link these two aspects to draw comprehensive conclusions. In contrast, this study used a comprehensive qualitative approach with data collected in interviews. This allowed us to pay attention to pilots’ real experiences, rather than constraining these experiences using set response options. Additionally, using focus group interviews meant that we could analyze pilots’ perspectives of challenges and support needs in relation to both physical and mental health aspects, which allowed a comprehensive analysis.

In this qualitative study, we hoped to offer preliminary mind mapping of pilots’ basic working conditions based on pilots’ experiences of their psychological and physical health through interviews. We aimed to analyze the experiences expressed by pilots in relation to various factors, such as energy and time distribution between work and life, exercise habits, diet and sleep regularity, pressure conditions, and the impact of the pandemic on work content and income. This allowed us to summarize pilots’ experiences of their profession, examine their perspectives of their own health, and explore their real support needs. The purpose of this series of qualitative focus group interviews was to clarify the challenges faced by pilots relating to both physical and mental health, and determine pilots’ needs and suggestions from the perspective of balancing physical and mental health. Ultimately, this study may provide guidance for follow-up intervention research to improve the relevance and effectiveness of strategies to improve pilots’ mental and physical health. Our overarching purpose was to provide pilots with more appropriate psychological and physical health support.

## 2 Materials and methods

Focus group interviews were carried out via the Tencent conference platform. Interviewed participants’ statements about their experiences on career-related topics were used as the data for analysis. COREQ guidelines were used to guide this study (12).

### 2.1 Study sample

A purposive sampling method was used to select pilots from nine civil aviation companies: two large, three medium-sized, and three small civil aviation companies and one cargo airline (which is a special type of civil aviation company). We randomly selected three or six pilots from each airline and conducted interviews in batches of three participants. The reason for selecting three participants for each focus group interview was that the privacy and duration of the interviews would have been difficult to control with more participants, and the information collected might have been incomplete with fewer participants. Participant selection was stratified by age and position (copilots: including pilot cadet and second copilot, and captains or squadron leaders) to cover all sub-groups of pilots. The inclusion criteria for this study were that participants must be actively employed as pilots and reside in East China. Any pilot that withdrew during the interview process was excluded from the valid sample. We considered the problem of information saturation, and the sample size and interview content were determined based on this principle. In total, we interviewed 42 pilots (Table 1). All participants provided informed consent before their interviews.

**Table 1:** Participants’ general information.

Serial number	Gender	age	Date of interview	Position	Serial number of company	Marital status	Record of formal schooling	Years of flight
1	Male	35	2022.8.8	Squadron leader	1	Married	Undergraduate course	13
2	Male	34	2022.8.8	Squadron leader	1	Married	Undergraduate course	11
3	Male	37	2022.8.8	Squadron leader	1	Married	Undergraduate course	15
4	Male	36	2022.8.9	Squadron leader	2	Married	Undergraduate course	14
5	Male	55	2022.8.9	Squadron leader	2	Married	Undergraduate course	18
6	Male	48	2022.8.9	Squadron leader	2	Married	Undergraduate course	22
7	Male	34	2022.8.9	Squadron leader	3	Married	Undergraduate course	6
8	Male	36	2022.8.9	Squadron leader	3	Married	Undergraduate course	8
9	Male	42	2022.8.9	Squadron leader	3	Married	Undergraduate course	14
10	Male	40	2022.8.10	Squadron leader	4	Married	Undergraduate course	14
11	Male	38	2022.8.10	Squadron leader	4	Married	Undergraduate course	13
12	Male	39	2022.8.10	Squadron leader	4	Married	Undergraduate course	13
13	Male	52	2022.8.10	Squadron leader	5	Married	Undergraduate course	18
14	Male	40	2022.8.10	Squadron leader	5	Married	Undergraduate course	18
15	Male	42	2022.8.10	Squadron leader	5	Married	Undergraduate course	17
16	Male	30	2022.8.10	Copilot	6	Married	Undergraduate course	3
17	Male	27	2022.8.10	Copilot	6	Unmarried	Undergraduate course	0.5
18	Male	30	2022.8.10	Copilot	6	Unmarried	Undergraduate course	3
19	Male	30	2022.8.11	Copilot	4	Unmarried	Undergraduate course	3
20	Female	29	2022.8.11	Copilot	4	Unmarried	Undergraduate course	3
21	Male	26	2022.8.11	Copilot	4	Unmarried	Undergraduate course	1
22	Male	43	2022.8.11	Squadron leader	7	Married	Undergraduate course	22
23	Male	45	2022.8.11	Squadron leader	7	Married	Undergraduate course	22
24	Male	43	2022.8.11	Squadron leader	7	Married	Undergraduate course	22
25	Male	32	2022.8.11	Copilot	8	Married	Undergraduate course	6
26	Male	31	2022.8.11	Copilot	8	Married	Undergraduate course	3
27	Male	27	2022.8.11	Copilot	8	Unmarried	Undergraduate course	3
28	Male	47	2022.8.15	Squadron leader	9	Married	Undergraduate course	23
29	Male	42	2022.8.15	Squadron leader	9	Married	Undergraduate course	20
30	Male	46	2022.8.15	Squadron leader	9	Married	Undergraduate course	21
31	Male	40	2022.8.16	Squadron leader	7	Married	Undergraduate course	19
32	Male	39	2022.8.16	Squadron leader	7	Married	Undergraduate course	15
33	Male	40	2022.8.16	Squadron leader	7	Married	Undergraduate course	19
34	Male	28	2022.8.16	Copilot	9	Unmarried	Undergraduate course	5
35	Male	25	2022.8.16	Copilot	9	Unmarried	Undergraduate course	5
36	Male	24	2022.8.16	Copilot	9	Unmarried	Undergraduate course	3
37	Male	49	2022.8.17	Squadron leader	8	Unmarried	Undergraduate course	23
38	Male	42	2022.8.17	Squadron leader	8	Unmarried	Undergraduate course	17
39	Male	43	2022.8.17	Squadron leader	8	Unmarried	Undergraduate course	20
40	Male	39	2022.8.17	Squadron leader	6	Married	Undergraduate course	15
41	Male	42	2022.8.17	Squadron leader	6	Married	Undergraduate course	17
42	Male	38	2022.8.17	Squadron leader	6	Married	Undergraduate course	18

Participants belonging to the same company were allocated to the same interview group, with each group comprising three participants. Overall, 14 groups were formed and interviewed successively.

### 2.2 Ethical considerations

This study was approved by the Ethics Committee of Civil Aviation Shanghai Hospital (no. 2021-7). All ethics procedures and governance requirements were adhered to for each interviewed participant. All participants were advised that participation was voluntary, they had the option to withdraw from the study, and the interview session could end at any time they requested without any repercussions.

### 2.3 Data collection

The interview outline was developed by the research team after reviewing relevant literature (13, 14), and reflected our purpose of improving pilots’ physical and mental health. The outline focused on participants’ working experience, living habits, and challenges in work and life. To optimize timely data collection, the research team conducted simulated interviews with two non-aviation practitioners and then modified the interview outline as necessary to avoid questions perceived as intruding on participants’ privacy or that had unclear meanings.

#### 2.3.1 Final interview outline

Q: You mostly work in the cabin of an airplane. Do you feel that it interferes with your leisure and family life? Are there fewer opportunities to exercise?

Q: In terms of diet, are your main meals served by the crew? Do you get bored and want something else for a change?

Q: It appears that your working hours are largely limited by the flight schedule. Is this a stressful working mode? What is your schedule like? How do you feel about your sleep? Have you experienced a sleep disorder (e.g., difficulty falling asleep, waking up at night, waking up early in the morning)?

Q: Does the stress from work interfere with your daily life?

Q: In the past 2 years, the airline industry was greatly affected by COVID-19. What were the differences between your working pattern during that period and non-COVID-19 times? Has the pandemic affected your income? Do you feel more stressed and anxious?

Q: In such a unique period, did you have a different view of work than before? Could you share your opinion on the measures taken by your country or your company for COVID-19 prevention, protection, and quarantine management? Does the company provide psychological counseling and other interventions? How does it work?

#### 2.3.2 Research protocol

Before the interviews, one researcher (M.X.) had preliminary contact with participants through WeChat and invited them to participate in the online focus group interviews via the Tencent conference platform. Only the research team members and participants attended the group interviews. Before each interview started, the interviewers introduced the research team members (including the name, identity, and responsibilities of each member) and the purpose of this qualitative research. The research team’s commitment to confidentiality of the interview data was also clearly stated. Consent was then obtained from all participants. All interviews were conducted in Chinese and then translated and reviewed by W.X. and Y.B. for inclusion in this manuscript.

#### 2.3.3 Interview process and reflection

As most participants were male, the interviews were mainly conducted by two male interviewers (Y.B. and H.H.), supplemented by questions from the supervising researcher. During interviews, the interviewers and participants turned on their cameras and enabled voice communication. The interviews were recorded to facilitate the subsequent arrangement of the interview results. Each interview lasted 45–90 min.

To improve the interview quality and ensure information saturation, members of the interview team analyzed and reflected on the problems encountered in the interview process and put forward practical suggestions after each interview.

### 2.4 Data analysis

Step 1: after the interviews, the researchers immediately transcribed the recordings. Colaizzi’s 7-step method was used for data analysis (15). We repeatedly read the interview materials to understand the general meaning.

Step 2: we extracted statements of significance and then coded recurring, meaningful ideas.

Step 3: we summarized the encoded ideas, which was followed by writing a detailed description without omissions.

Step 4: we then refined similar views and developed the theme concept.

Step 5: the analysis results were returned to participants for validation. In cases of disagreement, the research team resolved problems through discussion to ensure the accuracy of the results.

## 3 Results

Forty-two pilots from nine civil aviation companies (two large civil aviation companies, three medium-sized civil aviation companies, three small civil aviation companies, and one cargo airline) participated in the interviews. Most participants were captains (71%), and the remainder were copilots or flying cadets (29%). The majority of participants were aged over 35 years (64%), male (98%), married (71%), and had over 10 years of flying experience (67%). All participants had completed their undergraduate education (Table 2).

**Table 2:** Participants’ demographic characteristics.

Characteristics	Numbers	Percentage
Sex		
Women	1	2%
Men	41	98%
Age, years		
<35	27	64%
≥35	15	36%
Marital status		
Married	30	71%
Unmarried	12	29%
Record of formal schooling		
Undergraduate course	42	100%
Position		
Captain	30	71%
Copilot	12	29%
Years of flight, years		
<10	14	33%
≥10	28	67%

We identified three major themes from the interview data that covered the main types of issues reflected in participants’ narratives and the magnitude of their impact: (1) psychological stress; (2) physical health; and (3) pilots’ needs and suggestions.

### 3.1 Psychological stress

#### 3.1.1 Imbalance between family life and work

Some participants expressed that their stress originated from the lack of work-family balance. Pilots faced long-term absences from their families because one cycle of their flying assignments usually took several days (or more), and each interval between flying tasks was relatively short.

“Even after returning home, I may have to start a new round after several days, and the number of days I can return home might be only dozens of days in a year. In these days, there must be a lot of things to deal with. For people around our age, the biggest worry is the health of the older adults and children. Once they get sick, you can only rely on your spouse to take care of the four older adults and one child; even some second-child families will have two children, which is a greater pressure for the spouse. Some of our colleagues’ spouses will quit their jobs to be stay-at-home mothers for the sake of their families, which is also unfair to their spouses. They give up their careers for the sake of their families.” (male, 36 years, captain)

#### 3.1.2 Pressure from assessment and physical examination eligibility requirements

Some participants who were flying cadets found it difficult to complete their assessment to a high standard. The fear of making mistakes created major pressure, which resulted in serious psychological stress in the long term.

“When you’re in the cadet stage, the standard might be asking you to finish 40% of the job correctly, but the faculty is asking you to finish 60% of it correctly. They themselves might be able to do 90%, or even 100% of the job, without any mistakes. However, this is actually very difficult for us. Suppose you want to do 100 things, to make 10 to 20 mistakes is very easy, but if you allow yourself to make only one mistake, or not make any mistakes, it ends up being very difficult.” (male, 26 years, copilot)

Some participants emphasized that the physical examination, which was an essential part of their yearly assessment, also caused psychological stress. This was because failure to meet the physical examination standards implied a potential significant safety risk, which would directly result in suspension of that pilot’s medical certificate.

“We have a hard target to meet in our physical examination every year. If we fail to meet the target, our work arrangement will be affected.” (male, 30 years, copilot)

Given the stress caused by the various assessments, pilots reported experiencing a heavy psychological burden, especially older pilots. With all these stress from the various assessments, pilots reported heavy psychological burden, especially in older pilots. This finding provides a theoretical basis for the development of intervention to improve the mental health of pilots.

#### 3.1.3 Pressure caused by worries about being infected with COVID-19

Most participants reported that the COVID-19 pandemic had a huge influence on the civil aviation industry and on pilots themselves. They noted that the pressure experienced during the COVID-19 pandemic originated from the economic pressure caused by the decreased flying time, continuous isolation, and fear of contracting the virus.

“Meanwhile, the flights have reduced a lot since the COVID-19 pandemic, and now about half of them are cancelled. Many captains have a lot of pressure regarding mortgage payments especially during the lockdown period. Now, to most of the young people, even the captain, especially those whose home is not here in Shanghai, the pressure is rather big. There are so many family members that they need to take care of, parents, children, and so on. They could probably handle it before the pandemic, but now they lack confidence.” (male, 48 years, captain)

“Another point causing anxiety for our pilots is COVID-19 because we have to stop flying immediately when we are found to be infected in the physical examination. We have to wait until the nucleic acid test is negative, then be observed on the ground for 2 or 3 months and wait for the Civil Aviation Administration of China (CAAC) to make a decision. The whole process may last for 6 months. (…) Though we’re all under close management, and we’ve all taken precautions, Omicron is highly contagious with many variants, so there’s still a lot of risk. And if we get infected, we stop flying, which makes us very anxious.” (male, 36 years, captain)

### 3.2 Physical health

#### 3.2.1 Nutrition deficiency during working hours

Most participants shared that prepared airline meals during flight tasks created a serious problem regarding nutrition deficiency. They noted that this problem originated from three phenomena: irregular meal timings, imbalanced nutrition structure of prepared airline meals, and limited choice.

Many participants reported they had an irregular diet during flight tasks, which was closely related to the flight and landing times. This made it difficult for pilots to get sufficient nutrition when needed, which may harm their health.

“The total life schedule of our occupation is irregular because it is based on the time of the flight. For example, there are early or late flights sometimes. Under such circumstances, maybe some of the pilots do not follow the schedule in terms of diet. (…) For us, especially when the flight time is right around lunch time or dinner time, what we can do is simply postpone the time to eat or finish having meals in an extremely short time.” (male, 36 years, captain)

Most participants claimed that the nutritional structure of the airline meals could not be guaranteed. Although basic nutritional factors (e.g., the amount of energy, vitamins) were considered carefully when the meals were prepared to ensure that the pilots were well-conditioned and make the flight safe, the quality of the meals varied from company to company. Some participants reported high levels of salt and oil in their airline meals.

Many participants reported a tendency to overeat when finishing a flying task. Participants’ narratives emphasized that they tended to choose unhealthy foods as a kind of “compensation” for the prolonged intake of bland foods during flying tasks.

“After landing, most people really will go and overeat, and will be more likely to eat some food with strong flavor, such as spicy hot pot. Most of us know very well that this food is not healthy, but we really want to eat it.” (male, 27 years, copilot)

Some participants added that the limited choice of airline meals was a major problem for many pilots with allergies. For example, if airline meals happened to include allergens, most pilots had no choice but to skip the meal, which resulted in inadequate energy and nutrition intake, which could potentially damage the digestive system in the long term (16).

“There are a lot of people who are lactose intolerant, or allergic to legumes or other foods, so for those people there is little choice.” (male, 28 years, copilot)

Some participants expressed that it would benefit their physical and psychological health if the airline meals were more balanced in terms of nutrition. A similar request was also made in relation to meals provided during isolation periods because of the COVID-19 pandemic, as meals during quarantine were often provided by the airline for safety reasons. Moreover, some participants noted that more personalized meals or more choices in the daily meals may increase their desire to eat airline meals, which would prevent the lack of nutrition resulting from reluctance to eat airline meals. Several participants advised that the taste of the airline meals could also be improved to make pilots more willing to eat these meals to guarantee sufficient nutrition intake.

“All the quarantine hotels are short of vegetables. Because of the epidemic, we cannot buy materials from the outside, so we hope the company can intervene to provide us with some vegetables. Now there is an imbalance between meat and vegetables, and there is not much choice.” (male, 28 years, copilot)

#### 3.2.2 Changes in eating habits because of the COVID-19 pandemic

Some participants emphasized that their diet during the COVID-19 pandemic differed from that before the pandemic. On the one hand, the decreased flying time meant that pilots had more time to spend at home, which resulted in the normalization of daily diets. On the other hand, there were periods of isolation after finishing flights. The meals during this period were supplied by the company and were the same as the prepared airline meals, so there were still problems such as nutrition imbalance and limited choice.

“During the period of isolation, we mainly ate boxed lunches from the hotel, which was also arranged by the company. Some of them were greasy and not that healthy.” (male, 48 years, captain)

#### 3.2.3 Sleep deprivation

Although some participants said that sleep problems were within their range of self-regulation, about a half of the participants acknowledged that sleep deprivation often occurred, especially on morning and evening flights.

“There will be some cases of insomnia more or less. (…) They may sleep at home during the day before the flight so that they have more energy at night, but later they find that it is not appropriate as they can’t sleep in the morning after the night flight. Then some people will stay up in the morning, so that they can sleep better at night, but they still can’t make up for the lost sleep.” (male, 42 years, captain)

Some participants from large civil aviation companies with international airlines also reported that jet lag was a major problem which affected the quality of their sleep (17).

“The body is used to it. It can’t sleep when it’s time to sleep. It can’t rest when it’s time to rest.” (male, 52 years, captain)

#### 3.2.4 Occupational diseases

Participants expressed that the damage to their physical health originated from multiple occupational diseases. Most interviewed pilots had lumbar spondylosis and cervical spondylosis, which they attributed to sitting for too long and the discomfort of the cockpit seat. Several participants believed that working at high altitude for long periods of time could lead to various diseases such as heart disease, attributed to changes in radiation and air pressure at high altitude. A minority of participants reported they suffered from conditions such as hair loss, lithiasis, and high blood pressure, which may be closely related to dietary limitations during flight assignments. Two pilots reported that they suffered from shoulder arthritis, migraines, bloating, and debilitating mental problems. A female pilot also reported delayed menstruation.

“It’s not just sitting, it’s related extension issues, like cervical spondylosis, noise, that actually have a physiological impact all the time.” (male, 42 years, captain)

“The relative low pressure of the upper air and internal and external pressure difference may affect viscera organs and ear pressure.” (male, 46 years, captain)

“High blood pressure, lithiasis and so on may have something to do with flying, because for a long time on the plane we just sit there, rarely get up and do some activities, which may be some objective factors. It is hard to say is directly caused, but also have to say that this will bring about physical symptoms.” (male, 55 years, captain)

“After working for a certain time, sometimes you will find that menstruation becomes delayed or irregular.” (female, 29 years, copilot)

### 3.3 Pilots’ needs and suggestions

#### 3.3.1 Lack of support from the company in coping with stress

Most participants claimed that they experienced difficulty in releasing their psychological stress and required a workable approach to handle this stress. Participants mentioned that they worried about the impact on their career if they used psychological support provided by the company, which reduced the effectiveness of that support. That is, pilots wanted support options that did not involve the company. Participants noted that pilots tended to have a relatively small circle of friends, mainly limited to family and colleagues, so it was hard for them to find someone to talk to when dealing with psychological stress. In short, most participants expressed a desire for more chances to communicate and release their pressure without worrying that their complaint would become known to the company and therefore influence their career. This support was expected to be provided by a third party in the context of wider society.

“Many people may only be able to relieve stress through self-adjustment or through some communication with their regular colleagues. (…) Human beings are social animals. I think if we can have more opportunities to communicate and express our pressure, the effect may be better.” (male, 45 years, captain)

#### 3.3.2 Pilots’ yearly examination standards

One participant noted that the standards for physical examination were still the same as they were decades ago, which did not reflect the changes in public health. Therefore, he suggested updating the physical examination standards based on pilots’ current general health condition.

“The physical examination standards for pilots are still the same as they were 30 or 40 years ago. Considering the human health index has changed generally now, the standards should be adjusted appropriately as well.” (male, 47 years, captain)

#### 3.3.3 Support with sports equipment

Some participants reported a need for financial support to buy sports equipment, as the sports/activity facilities supported by the company often took too much time to access. Some participants advised that the company could try different methods such as providing gym cards or sports equipment vouchers.

“The company has some measures anyway. There are gyms, physical therapy rooms, and so on but you have to use your commute time to get there from home.” (male, 34 years, captain)

“I hope the company can reimburse a little sports equipment every year, such as for basketball, volleyball, and so on.” (male, 34 years, captain)

#### 3.3.4 Respect planned rest time

Participants noted the need for more rest time. Most companies had adopted the “2-day rest after 3-day flight” pattern, but participants explained that during the “2-day rest” period, they were required to handle extra work such as flight preparation and some administrative work. They expressed that the company required them to fly as often as possible to earn more benefits to the company, which resulted in high fatigue conditions. Vacations rostered by the company were usually inadequate for them to recover. However, because of their income, they did not want to take extra leave to get a thorough rest. Therefore, they hoped that they could have more paid vacation time that could give them the chance to relax completely.

“We usually fly for 3 days and then have a rest for 2 days, but we still have to go to work at the company during these two days, because we still have a lot of groundwork and administrative work to do, so it is still a busy situation. Sometimes I feel tired, but I still have to fly for 3 days. After all, I don’t get paid if I don’t fly.” (male, 47 years, captain)

“We hope that we can learn something from Western management. Western countries have a system of paid vacation. It seems very insignificant, but people can be completely relaxed in this situation.” (male, 40 years, captain)

#### 3.3.5 Isolation periods

Moreover, as our interviews were conducted during the COVID-19 pandemic period under a strict isolation policy, most participants reported long isolation periods after each flight, which often lasted for 2 weeks to several months. These periods of isolation during the COVID-19 pandemic were considered too long. Many participants stated that the isolation could be shortened as the long-term isolation directly led to their anxiety.

“I suggest that the isolation period be reduced appropriately and the 4+3 mode (i.e., 4 days of centralized isolation and 3 days of home isolation) be adopted.” (male, 48 years, captain)

## 4 Discussion

In this qualitative study, participating pilots described various psychological and physical health challenges. These challenges had implications for pilots’ well-being and at last flight safety.

### 4.1 Psychological health

In our study, participants reported multifactorial stressors related to the absence from their family, assessments, and the COVID-19 pandemic. The main stressors among pilots varied across different age groups. Stress among younger pilots mainly originated from their financial situation, promotion opportunities, and social interactions, whereas that among older pilots mainly arose from concerns about their health and being absent from their families. The differences in stressors between older and younger pilots may be because an individual’s self-perception of aging and their physical condition becomes worse with age, which in turn leads to deterioration of health (18). In addition, older pilots tended to attach more importance to family, meaning that their absence from family life became a significant source of stress. Our results were consistent with some previous studies that also emphasized that stress among younger pilots mainly resulted from worries about income and promotion and led to mental health issues (19, 20). Conversely, mental health issues among older pilots tended to originate from being absent from their families. A previous study highlighted the importance of being accompanied by family members to improve mental health (21). In addition, a study focused on stressors experienced by Chinese pilots highlighted that the risk of income reduction, contracting COVID-19, stress at work, and the risk for a delayed upgrade schedule were major mental health stressors during the COVID-19 pandemic (20).

Various promising methods could be considered by civil aviation companies to improve pilots’ mental health. For example, given the financial pressure pilots faced during the COVID-19 pandemic because of reduced flights, airlines could provide pilots with more care focused on daily life (20, 22). In addition, measures to guarantee pilots’ family security may offer a promising solution to relieve mental health pressures caused by long periods of absence from their families during the COVID-19 pandemic, such as providing subsidies to families for daily necessities during pilots’ isolation periods. Participating pilots mentioned that various limitations still existed despite civil aviation companies attempting to improve staff mental health, such as by establishing psychological counseling rooms. Although most companies had a counseling room, most pilots preferred not to use these services because they feared doing so would negatively affect their future careers. Instead of psychological support directly provided by the companies, social support may provide an effective solution to improve pilots’ psychological health, which was demonstrated in a previous study (23). Economic support for pilots’ careers and families and some appropriate social support may also help to relieve their stress, thereby improving pilots’ mental health, especially during the COVID-19 pandemic.

### 4.2 Physical health

Our findings highlighted that a poor diet, poor sleep quality, and occupational diseases were the main factors affecting pilots’ physical health based on their self-report. Participants commented on the irregular and high-salt diet when completing flight tasks or isolation periods, which they believed was harmful to their physical health. Research shows that irregular diets can increase the risk for esophageal and stomach cancer (22). The high-salt diet reported by some participants increased the possibility of high blood pressure, cardiovascular disease, and stroke (24). Sleep problems are common among pilots; for example, a previous survey found 24.6% of short-haul and 23.5% of long-haul pilots reported major sleep problems 8 nights a month (3). Furthermore, previous studies found that pilots’ health problems were mainly in the cervical, shoulder, and lumbar spine, such as cervical spondylosis, periarthritis of the shoulder, reflex shoulder, arm and hand pain, swelling and numbness, back and leg pain, lumbar disc herniation, and lumbar muscle strain (10, 25–28). These factors were also reported in our interviews. In addition to those diseases, our participants reported problems such as hyperlipidemia, hypertension, dyspepsia, lithiasis, endocrine dysfunction, and neurasthenia.

The emphasis on physical health problems reported by different age groups also differed. Generally, younger pilots reported more cases of diet problems, whereas older pilots reported more sleep problems. Occupational diseases were common among pilots but more pronounced among older pilots, which was consistent with the results of a previous study (19). This may be because most occupational diseases that pilots are prone to are chronic (29). For example, a study on lipid status among pilots found that high triglycerides and total cholesterol levels among pilots were related to older age and longer flight time (30).

From a physical health perspective, most participants expressed an urgent need for more choice of meals and more rest time. It is worth noting that companies should place more consideration on the cost of welfare measures (e.g., time and convenience), which may influence whether pilots are willing to use these measures. For example, the company could organize regular physical examinations for cardiovascular problems, which are common among pilots, and offer medical care or convalescent leave to pilots with poor results. Moreover, airlines could design reasonable and scientific flight schedules to guarantee sufficient sleeping time. More attention should also be paid to nutritional composition when preparing airline meals.

### 4.3 Implications

Our results have some practical implications that may help civil aviation companies and air transport management departments to improve pilots’ physical and psychological health. Currently, the aviation medical field lacks a scientific and comprehensive assessment method for civil aviation pilots (31–33), including factors such as physical health, mental health, and healthy behaviors, as well as a dynamic monitoring platform and health management system for health data in this field. Many physical or psychological assessment methods conducted for individuals or other occupational groups are not suitable for pilots because pilots receive special learning and training, and their working conditions differ from those to which others may be exposed (31, 32). Furthermore, relatively little research has been conducted on civil aviation pilots’ physical and mental health, with a notable lack of high-quality research (20, 34, 35). Most available studies were based on scales, questionnaires, or standardized questions (1–6). Therefore, we attempted to capture pilots’ perspectives of their physical and mental health-related challenges and needs through face-to-face conversations via qualitative interviews. This allowed us to confirm information or seek clarity during the interviews to ensure accuracy. We comprehensively collected the information provided by participants during the interviews and paid equal attention to each participants’ personalization, meaning our results are rich and specific. Our study will provide evidence to inform the development of a more scientific and comprehensive physical and mental health evaluation system for civil aviation pilots. This will help to achieve “early detection, early prevention, early intervention, and early treatment.” Through the development of all-round, multi-dimensional, and different life cycle stages of “health portraits,” it is possible to promote safer management of civil aviation pilots’ physical and mental health and reduce the occurrence of physical and mental health damage.

### 4.4 Limitations

There were some limitations in this study. First, because of the serious imbalance in the male to female ratio in the occupational group of pilots, only one female participant was interviewed; therefore, our study might not be representative of the experience of female pilots. However, only 0.44% of airplane pilots in China are women, as reported in the Annual Report of Chinese Civil Aviation Pilot Development (36); therefore, our study provided relatively robust evidence regarding the challenges of pilots’ psychological and physical health. Second, because the interviews were mainly conducted in groups, some questions, especially those that touched on participants’ privacy, could not be explored in-depth. A further study using individual interviews is needed to resolve this issue. Third, the purpose of our study was to investigate pilots’ health conditions; therefore, comparisons among companies were relatively weak and more evidence is needed. For example, we did not explore the differences in welfare benefits between companies, so we could not determine the relationship between differences in these benefits and pilots’ various needs. We intend to conduct further research in this area. Finally, our study investigated pilots’ health challenges and needs through focus group interviews. Although this method meant that personalization was emphasized compared with questionnaire surveys, the results could not be quantified, and it was not possible to analyze the correlations between various factors and the results or the correlations between various factors. In addition, this study was based on self-reported information, and the results obtained may be biased because of individual differences in expression. Obtaining accurate data (e.g., physical examination results) was also difficult. Therefore, we need to combine other research methods in a further study to improve the accuracy of the results.

## 5 Conclusion

In this study, we interviewed 42 pilots and found that they experienced a large amount of psychological pressure from various factors. Their physical health condition was also worrying, and pilots had several suggestions in relation to improving their physical and mental health. Although pilots are generally satisfied with the working environment, they are aware that the schedule and content of their work can have a negative impact on their physical and mental health. In addition, the COVID-19 pandemic situation and the lack of suitable professional assessment are also health-related problems that remain to be resolved. Civil aviation companies, air transport management departments, and aviation hospitals pay insufficient attention to pilots’ physical and mental health. Therefore, it is necessary to establish a systematic, scientific, and personalized medical intervention system for pilots going forward. Through this qualitative study, we identified pilots’ perspectives of challenges and support needs in relation to psychological and physical health, and also laid a foundation for subsequent research. We hope to attract more researchers to conduct comparative studies of standard health measures for pilots in different parts of the world. This will provide a reference for civil aviation companies and governments worldwide to support improvement of pilots’ working conditions, which will ultimately improve pilots’ physical and mental health and contribute to overall aviation safety.

## Data availability statement

The raw data supporting the conclusions of this article will be made available by the authors, without undue reservation.

## Ethics statement

The studies involving humans were approved by the Ethics Committee of Civil Aviation Shanghai Hospital (no. 2021-7). The studies were conducted in accordance with the local legislation and institutional requirements. The participants provided their written informed consent to participate in this study. Written informed consent was obtained from the individual(s) for the publication of any potentially identifiable images or data included in this article.

## Author contributions

WX: Data curation, Writing – review & editing, Writing – original draft. YB: Data curation, Writing – review & editing, Writing – original draft. LZ: Data curation, Writing – review & editing. YL: Data curation, Writing – review & editing. EZ: Data curation, Writing – review & editing. HL: Data curation, Writing – review & editing. QJ: Data curation, Writing – review & editing. YC: Data curation, Writing – review & editing. QD: Data curation, Writing – review & editing. FS: Data curation, Writing – review & editing. LW: Data curation, Writing – review & editing. ZL: Data curation, Writing – review & editing. XC: Data curation, Writing – review & editing. QG: Data curation, Writing – review & editing. HH: Data curation, Writing – review & editing. BR: Data curation, Writing – review & editing. YS: Data curation, Writing – review & editing. MX: Data curation, Supervision, Writing – review & editing, Investigation, Project administration.
